# Dietary Phenolic Compounds as Anticancer Natural Drugs: Recent Update on Molecular Mechanisms and Clinical Trials

**DOI:** 10.3390/foods11213323

**Published:** 2022-10-23

**Authors:** Saad Bakrim, Nasreddine El Omari, Naoufal El Hachlafi, Youssef Bakri, Learn-Han Lee, Abdelhakim Bouyahya

**Affiliations:** 1 Geo-Bio-Environment Engineering and Innovation Laboratory, Molecular Engineering, Biotechnology, and Innovation Team, Polydisciplinary Faculty of Taroudant, Ibn Zohr University, Agadir 80000, Morocco; 2 Laboratory of Histology, Embryology, and Cytogenetic, Faculty of Medicine and Pharmacy, Mohammed V University in Rabat, Rabat 10100, Morocco; 3 Microbial Biotechnology and Bioactive Molecules Laboratory, Sciences and Technologies Faculty, Sidi Mohmed Ben Abdellah University, Fes 30000, Morocco; 4 Laboratory of Human Pathologies Biology, Department of Biology, Faculty of Sciences, Mohammed V University in Rabat, Rabat 10106, Morocco; 5 Novel Bacteria and Drug Discovery Research Group (NBDD), Microbiome and Bioresource Research Strength (MBRS), Jeffrey Cheah School of Medicine and Health Sciences, Monash University Malaysia, Subang Jaya 47500, Malaysia

**Keywords:** cancer, dietary phenolic compounds, apoptosis, clinical trials

## Abstract

Given the stochastic complexity of cancer diseases, the development of chemotherapeutic drugs is almost limited by problems of selectivity and side effects. Furthermore, an increasing number of protective approaches have been recently considered as the main way to limit these pathologies. Natural bioactive compounds, and particularly dietary phenolic compounds, showed major protective and therapeutic effects against different types of human cancers. Indeed, phenolic substances have functional groups that allow them to exert several anti-cancer mechanisms, such as the induction of apoptosis, autophagy, cell cycle arrest at different stages, and the inhibition of telomerase. In addition, in vivo studies show that these phenolic compounds also have anti-angiogenic effects via the inhibition of invasion and angiogenesis. Moreover, clinical studies have already highlighted certain phenolic compounds producing clinical effects alone, or in combination with drugs used in chemotherapy. In the present work, we present a major advance in research concerning the mechanisms of action of the different phenolic compounds that are contained in food medicinal plants, as well as evidence from the clinical trials that focus on them.

## 1. Introduction

Cancer is a complex disease that is linked to several risk factors, such as bacterial and viral infections, oxidative stress, genetic mutation, poor nutrition, and epigenetic disturbance. The transformation mechanisms range from genetic and hormonal disturbances to environmental inducers and metabolic deregulations. This divergence of risk factors gives rise to various forms of cancer, and sometimes, implies therapeutic specificity even for the same type of cancer [1,2,3]. In this regard, the search for anticancer treatments requires the screening of several chemical molecules with functional diversity. Among the candidate molecules studied, we found phenolic compounds. These compounds are recognized for their extensive pharmacological properties, such as anti-inflammatory, antibiotic, antiseptic, antitumor, antiallergic, and cardioprotective properties, among others. In this context, edible medicinal plants are considered to be a very important source of anti-cancer molecules. Indeed, polyphenolic compounds such as flavonoids and acid phenolics are of considerable relevance as anti-cancer drugs [4].

Phenolic compounds are derived from edible plants, particularly medicinal and aromatic plants, which are found in many food products such as vegetables, cereals, legumes, fruits, nuts, and certain beverages. In fact, this chemical family constitutes a group of substances frequently present in the metabolism of medicinal plants, and it contains several subclasses such as phenolic acids, flavonoids, and tannins, which are the most abundant molecules [5,6,7]. These compounds have chemical functional groups that allow them to target the different signaling pathways involved either in cell transformation or in the promotion of cell transformation.

Various investigations have focused on phenolic compounds as anticancer drugs. As a matter of fact, these groups of molecules exert anticancer properties by acting on the multiple checkpoints of cancerous cells, and they can induce apoptosis, autophagy, and cell cycle arrest with high specificity [8,9]. In addition, phenolic compounds exert different mechanisms such as inhibiting telomeres, blocking their expression and inhibiting angiogenesis and metastases [10,11]. These compounds induce remarkable effects against human cancers by acting on molecular targets, notably by reducing the expression of a transcription factor regulating the expression of cytoprotective genes, reducing p53 activation, decreasing Bcl-2 expression and mitochondrial membrane potential, suppressing the expression of HIF-1α, and increasing cellular apoptosis with the downregulation of p-Akt expression [10,11]. Based on previous discussions, this present investigation aims to highlight the potential benefits of dietary phenolic compounds in managing and preventing human cancer; therefore, in this work, we have presented a major recent advance in research concerning the different molecular mechanisms of the substances that contain phenolic compounds, as well as the clinical trials that are carried out on these natural substances which target different human cancers.

## 2. Research Methodology

A comprehensive systematic review was conducted to highlight all relevant in vivo, in vitro, and clinical research on the potential use of dietary phenolic compounds, and their major secreted secondary metabolites, in order to study their molecular pathways for application as potential natural anticancer drugs. We consulted different credible repositories and scientific databases, such as ScienceDirect, Scopus, Springer Link, Wiley, Web of Science, Google Scholar, and PubMed. Recent comprehensive papers in peer-reviewed publications up to April 2022 were considered. There was no time limit on the year of publication. Only articles written in English were inventoried and used in this article. Exclusion criteria for unscreened articles comprised letters to the editor, non-English language publications, unpublished reports, and conference abstracts. The search strategy was performed using several keywords included within the literature quest, such as ‘Dietary phenolic compounds’, ‘Phenolics compounds and nutraceutical’, ‘Phenolics compounds and drugs’, ‘Phenolics compounds and targeted mechanisms’, ‘Polyphenols in cancer’, ‘Flavonoids in cancer’, ‘Stilbenes in cancer’, ‘Resveratrol in cancer’, ‘Curcumin in cancer’, ‘Phenolic acids in cancer’, ‘Caffeic acid in cancer’, ‘Gallic acid in cancer’, ‘Ellagic acid in cancer, ‘Sinapic acid in cancer’, ‘Rosmarinic acid in cancer’, ‘Flavonoids in cancer’, ‘Flavonols in cancer’, ‘kaempferol in cancer’, ‘Quercetin in cancer’, ‘Flavones in cancer’, ‘Apigenin in cancer’, ‘Genistein in cancer’, ‘Luteolin in cancer’, ‘Epigallocatechin-3-gallate in cancer’, ‘Anthocyanidins in cancer’, ‘apoptosis’, ‘in vitro’, ‘in vivo’, and ‘clinical trials’. A total of 138 peer-reviewed research papers published in the English language were included in this review.

## 3. Dietary Phenolic Compounds as Sources of Nutraceuticals and Drugs

Currently, an increasing level of attention is being placed on dietary components and their ability to aid the avoidance of certain risk factors that are involved in the genesis of complex pathologies; this is due to their high secondary metabolite content, which includes phenolic compounds. These bioactive agents have powerful antioxidant, anti-inflammatory, and anti-tumor properties, and consequently, they constitute key compounds for promoting healthiness and preventing diseases, especially cancer [12]. Polyphenols can be divided into different categories according to the number of phenolic cycles and organizational structures that bind these cycles to each other. Phenolic acids represent about one-third of the polyphenolic components found in food, and they comprise two principal groups: hydroxycinnamic acid derivatives (Caffeic acid, Coumaric acid, Ferulic acid, Sinapic acid) and hydroxybenzoic acid derivatives (Protocatechuic acid, Gallic acid, *p*-Hydroxybenzoic acid). Foods with a high phenolic acid content include kiwis, apples, coffee, tea, berries, pears, chicory, and cherries [13]. In addition, flavonoids are among the most common polyphenols consumed in the human diet, and over 4000 varieties of these substances have been recognized [14]. These natural components, which include seven subclasses (anthocyanins, flavonols, flavanols, flavones, flavan-3-ols, chalcones, and isoflavones), are commonly contained in red wine, red cabbage, cherries, black grapes, strawberries, and berries. Flavonols, including Stilbene and Myricetin, have been mainly found in blueberries, onions, leeks, kale, and broccoli. Other important dietary flavonoids are isoflavones, including Genistein; soy products and soybeans are food sources that have the richest levels of these compounds, given their estrogen-like structures [15]. Several phenolic compounds are directly implicated in the sensory characteristics of foods, and are therefore essential to their quality [16].

From the perspective of discovering efficient therapeutic options or combining treatment methods for fighting and preventing heavy and complex illnesses, as well as to guarantee optimum efficacy and outcomes, nutraceuticals are becoming a viable alternative to remedy many physiological and biological deficiencies. Nutraceuticals are described as a group of foods, though they can also be found in some drugs, that are characterized by their similarity to conventional foods, but they offer proven benefits in terms of physiology. Nutraceuticals are products obtained from foods, but they can also be used medicinally in the form of capsules, liquids, or pills, which also possess some physiological advantages. In recent decades, the use of these functional foods has gained considerable interest, and great advances have been made in terms of understanding their nutritional, therapeutic, and safety properties [17]. Recently, it was revealed that various natural bioactive molecules, such as phenolic compounds, exhibit anti-cancer activities, thus enabling them to destroy transformed or cancerous cells in a manner that is not harmful to their healthy homolog; however, this phenomenon appears to be associated with the use of natural ingredients in association with conventional drugs, thereby highlighting the possibility that supplementation with nutraceuticals may enhance the effectiveness of anticancer research and management strategies. Furthermore, nutraceuticals, or functional foods, may be promising candidates for reducing risk factors for cancer development due to their antioxidant and anti-inflammatory properties, and their ability to inhibit cell proliferation [18].

In fact, polyphenols, as phytochemicals consumed as a regular part of the diet (approximately 1 g/day) and available in great abundance in plant-based foods, especially fruits and vegetables, should be regarded as a source of nutrients that assists in the production of nutraceuticals, supplementary treatments, and drugs; therefore, they may be used as part of a new approach that aids the management of several diseases, including cancer.

The purpose of the following section is to highlight, from a mechanistic perspective, the prominent role of dietary phenolic compounds in the different processes that cause tumor transformation, development, and metastasis.

## 4. Dietary Phenolic Compounds as Anticancer Drugs: Targeted Mechanisms

**Figure 1:**
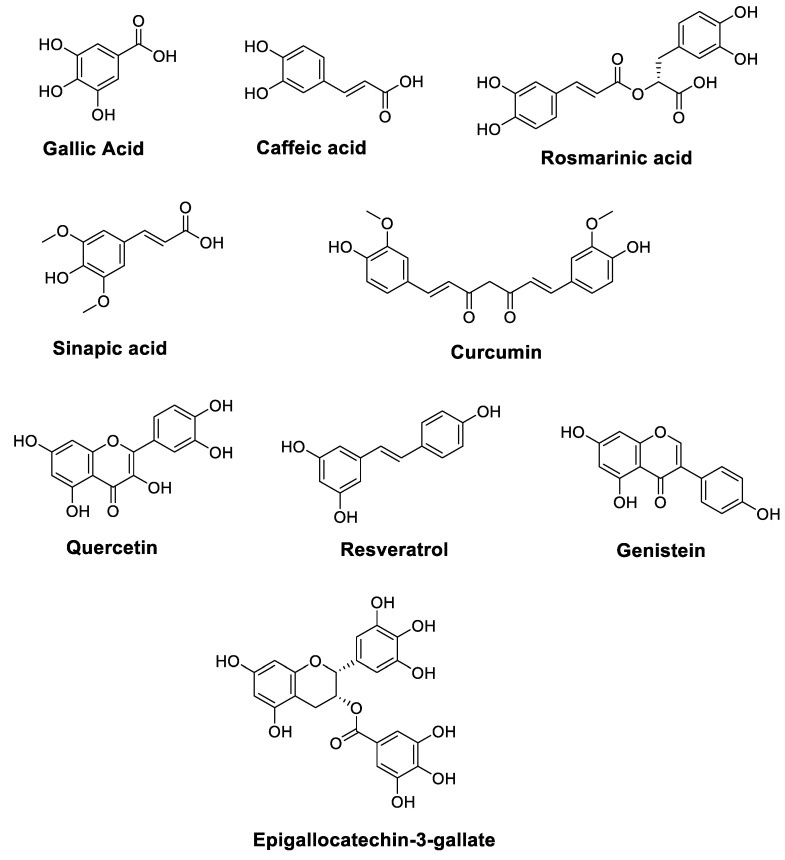
Chemical structures of polyphenols with anticancer properties.

Cancer is one of the leading causes of death worldwide, and it has become a largely preventable disorder with a significant response to modulation via nutritional factors [19,20]. The anticancer characteristics of vegetables and fruits have been partially associated with high levels of polyphenols. Figure 1 illustrates the chemical structures of polyphenols such as Resveratrol, Gallic acid, Caffeic acid, Rosmarinic acid, Sinapic acid, Quercetin, Genistein, Epigallocatechin-3-gallate, and Curcumin, which are essential components of the daily human diet, and they represent the predominant polyphenol content of foods. In addition to vegetables and fruits, bark, seeds, leaves, and flowers also provide abundant sources of polyphenols [21,22].

**Figure 2:**
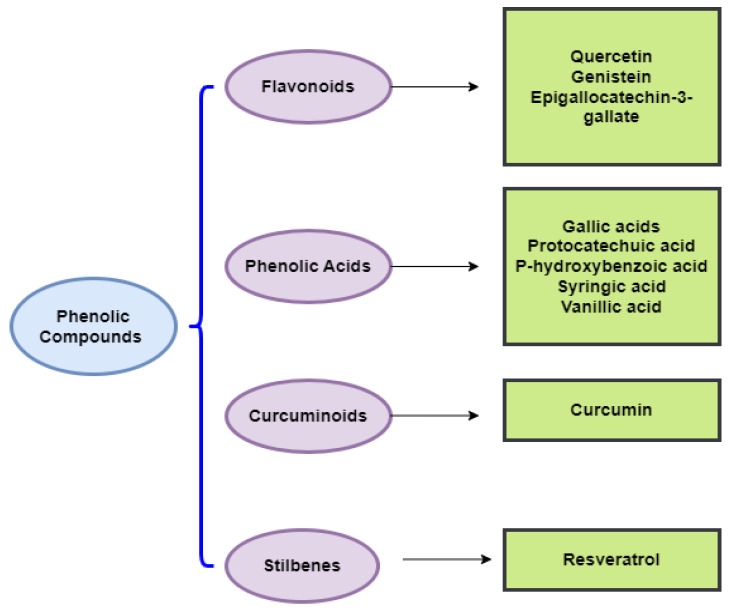
Chemical classes of dietary phenolic compounds exhibiting anticancer properties.

As conventional therapeutic and surgical approaches have failed to adequately manage various types of cancer, the need to identify and develop chemopreventive strategies has become a public health priority [20]. In this respect, the chemopreventive benefits of dietary polyphenols are principally attributed to their anti-angiogenic, cell cycle arrest, anti-metastatic, anti-inflammatory, anti-proliferative, autophagic, and apoptotic activities [20]. Indeed, dietary polyphenols can confer an indigenous defense by promoting cell signaling processes, including activator protein-1 (AP-1) DNA binding, activating the nuclear factor kappa B (NF-κB), phosphoinositide 3-kinase (PI3-kinases)/protein kinase B (PKB)/Akt pathway, biosynthesizing glutathione, and translocating nuclear factor 2 erythroid-related (Nrf2) into the nucleus, as well as mitogen-activated protein kinase (MAPK), extracellular signal-regulated proteinase (ERK), c-jun N-terminal kinase (JNK) (Figure 2), and P38 [23]. To assess the effectiveness of chemoprevention by dietary phytochemicals, the following molecular targets of polyphenols for the mechanistic regulation of chemoprevention pathways are discussed in more detail below. Listed in Table 1 are the dietary polyphenols that act as anticancer agents.

In the following sections, we will discuss the anticancer action of different chemical classes of phenolic compounds, in accordance with Figure 2.

### 4.1. Phenolic Acids

Phenolic acids are among the most common non-flavonoid plant phenolic components that exist as glycosides or aglycones (free form) [107]. They are widely distributed in plants and are found in oilseeds, grains, legumes, vegetables, fruits, herbs, and beverages. Hydroxybenzoic and hydroxycinnamic acids are two classes of phenolic acids based on C1–C6 and C3–C6 skeletons [108]. Hydroxycinnamic acids include *p*-coumaric, ferulic, caffeic, cinnamic, and sinapic acids. In contrast, hydroxybenzoic acids are represented by Gallic, Protocatechuic, *p*-Hydroxybenzoic, Syringic, and Vanillic acids [107]. Hydroxycinnamic and hydroxybenzoic acids, and their derivatives, exhibit high antioxidant and antiproliferative properties in vitro and in vivo by regulating different signaling pathways [109,110].

#### 4.1.1. Gallic Acid

Gallic acid (GA) (3,4,5-Trihydroxybenzoic acid) (Figure 1) exerts potent anticancer properties due to its remarkable antioxidant properties. In a recent study, using an oral squamous cell carcinoma (FaDu and Cal33) cell line, Gallic acid enhanced the anticancer efficacy of Docetaxel, Cisplatin, Doxorubicin, 5-FU, and Paclitaxel in combination with gamma irradiation in vitro, through the superoxide-mediated apoptosis pathway powered by lipophagy inhibition in an NRF2-dependent signaling pathway [31]. In vitro and molecular docking methods were used to investigate the anticancer potential of GA, which was isolated from a *Ceriops tagal* leaf methanolic extract, against two human cancer cell lines, HeLa (cervical) and MDA-MB-231 (breast) cancer cells. In fact, at 24 h, the IC_50_ values for GA were 4.18 ± 0.45 and 80.04 ± 0.19 μg/mL for HeLa and MDA-MB-231 cell lines, respectively. Moreover, molecular docking results revealed that GA exhibits an estimated binding free energy (ΔG) of −5.4 kcal/mol to the Bcl-B protein, the drug target. The estimated inhibition constant (Ki) for GA was 110 μM. This molecule may offer a considerable opportunity to block the overexpression of the cancer drug target protein in cancer cells [24].

Ko et al. carried out a recent investigation to examine the anticancer potential of GA against a non-small cell lung carcinoma (NSCLC) cell line A549 in vitro. The 48 h cell viability assay showed that GA treatment inhibited cell proliferation in a dose-dependent manner [25]. Additionally, the findings showed that the inhibition of the PI3K/Akt pathway was mediated by AG upregulation in p53 (tumor suppressor protein), which subsequently regulated intrinsic apoptotic proteins such as Bcl-2, Bax, and it cleaved caspase-3 and cell cycle-related proteins such as p27, p21, Cyclin E1, and D1, as evidenced by the Western blot analysis. In the in vivo model, AG treatment for four weeks reduced tumor mass size in the A549-derived tumor mouse model. Moreover, GA lowered the expression of p-Akt and the proliferating cell nuclear antigen and increased the expression of cleaved caspase-3 expression in tumor tissues.

GA is one of the major bioactive compounds in *Dovyalis caffra*. In this regard, the methanol extract of this plant was found to exert a potential anticancer action (58.90% toxicity) on HepG2 cells at 1000 µg/mL [26]. Furthermore, a recent study by Sanchez-Martin et al. showed that GA could be a promising agent in cancer prevention via its interaction with G-Quadruplexes. Results also revealed that GA exhibits a selective antitumor effect in colorectal cancer [27]. The IC_50_ values for CRL1790, SW480, and SW620 were >100, 22.39 ± 2.12, and 11.83 ± 1.54 µM, respectively. Moreover, results showed that GA-induced cell cycle arrest at the S and G_2_/M phases triggers nucleolar stress and downregulates G4-containing genes. In addition, as shown from immunofluorescence being used on BG4 antibodies, GA stabilized G4 in both in vitro and in vivo cellular environments, thus leading to tumor growth inhibition in colorectal cancer xenografts. These researchers also proved that GA binding to G4s is present in 5’ETS and the CMYC promoter.

Similarly, treatment of human prostate carcinoma DU145 cells with GA showed potent cell growth inhibition, apoptotic death, and cell cycle arrest in a dose- and time-dependent manner. It also showed the downregulation of cyclin- and cyclin-dependent kinases, and a marked induction of Cip1/p21. Other investigations indicated that GA causes Tyr15 phosphorylation early in cell division cycle 2 (cdc2). Furthermore, GA induced cdc25A and cdc25C phosphorylation, through ataxia telangiectasia mutated (ATM) and checkpoint kinase 2 (Chk2) activation, when DNA became damaged. Moreover, GA induced the cleavage of caspase-3, caspase-9, and poly (ADP)ribose polymerase [29]. Similarly, GA isolated from grape seed extract could be a promising phytochemical against prostate cancer. It exhibited the significant inhibition of growth and apoptotic death of DU145 cells in a dose- and time-dependent manner, and it caused caspase-9, caspase-3, and PARP cleavages [28].

Inoue et al. examined the cytotoxic effect of GA in different cell lines. These researchers indicated that the IC_50_ for mouse lymphoid neoplasm (P388- D1), human epithelial carcinoma (HeLa), human hepatoma (PLC/PRF/5), rat hepatoma (dRLh-84), human promyelocytic leukemia (HL60RG), and human epidermoid carcinoma (KB) are 4.8, 6.1, 6.6, 6.2, 5.4, and 13.2 µg/mL, respectively [32]. In contrast, the IC_50_ values for endothelial cells and fibroblasts were over 20 µg/mL. These findings indicate the ability of GA to promote cell death in tumor cells with comparatively high selectivity. Similarly, Sachithanandam et al. examined the anticancer potential of GA isolated from the *Ceriops tagal* leaf methanol extract against two human cancer cell lines, namely, HeLa (cervical) and MDA-MB-231 (breast) cancer cells. Indeed, at 24 h, the IC_50_ values were 99.91 ± 0.18 and 18.29 ± 0.12 µg/mL for the HeLa and MDA-MB-231 cell lines, respectively [24]. Molecular docking results reported that GA has an estimated binding free energy (ΔG) of −5.4 kcal/mol for the drug target protein Bcl-B.

#### 4.1.2. Caffeic Acid

Natural caffeic acid (*E*)-3-(3,4-Dihydroxyphenyl)prop-2-enoic acid (Figure 1) is well-known to possess several biological properties, including anticancer effects. In this context, Min et al. reported that CA significantly inhibits the growth of H1299 NSCLC cells by inducing apoptosis [41]. CA combined with Paclitaxel (PTX) provides a synergistic anticancer action against H1299 cells. Moreover, these researchers found that the combined treatment of PTX and CA induces a reduction in the proliferation of H1299 NSCLC cells. As evidenced by flow cytometry, CA treatment increased apoptosis, caspase-3 and caspase-9 activities, and the sub-G_1_ phase arrest of H1299 cells. In addition, CA increased the phosphorylation of kinase1/2 and the c-Jun NH2-terminal protein kinase1/2, and increased the PTX-induced activation of Bid, Bax, and downstream cleaved PARP. The combined treatment showed no significant side effects and strongly suppressed tumor growth in H1299 xenografts. Similarly, Kanimozhi et al. attempted to assess the molecular mechanism of CA as an anticancer agent against Hela and ME-180 cells [42]; therefore, CA increased markers of lipid peroxidation, such as connective dienes, reactive thiobarbituric acid substances, and lipid hydroperoxide. This molecule also enhanced morphological changes, altered mitochondrial membrane potential, and increased ROS levels in CA-treated cells, suggesting that CA possesses anticancer activity due to its pro-oxidant effect [42].

The results of a study by Bhat et al. suggested that CA has a pro-oxidant action through its involvement in the mobilization of endogenous copper, which is likely to be the copper bound to chromatin [43]. In fact, CA induced DNA disruption in human peripheral lymphocytes and produced oxidative stress in lymphocytes, which is suppressed by ROS scavengers and neocuproine [43]. Additionally, Guo et al. reported an easy synthesis of silver nanoparticles (AgNPs) using CA [44]. These particles exhibited potent anticancer activity. Data showed that AgNPs induce cytotoxicity in human hepatoma HepG2 cells in a dose-dependent manner; they could enter cells and inhibit tumor viability through the induction of apoptosis [44]. An investigation by Rosendahl et al. indicated that CA imitates anti-estrogen activity and alters key IGF-IR/pAkt and ER/cyclin D1 growth regulatory signals, thus leading to cell cycle damage and diminished cell proliferation [50]. CA showed anticancer activities against breast cancer ER+ and ER- that might raise tumor cells’ sensitivity to tamoxifen and diminish breast cancer growth [50]. Chen et al. conducted a study to evaluate the impact of CA on the toll-like receptor 4 (TLR4) signaling pathway. CA reduced NF-κB activation and IL-12 synthesis [51]. Nevertheless, it impaired the TLR4 pathway by altering the TLR4/MD2 complex. Results showed that the downregulation of the expression of TLR4, TRIF, and IRAK4 leads to apoptosis in breast cancers [51].

Research findings revealed that CA prevents cancer cell progression by blocking the histone demethylase (HDM) gene. CA suppressed DNA methylation catalyzed by prokaryotic DNA methyltransferase (DNMT) M.SssI and human DNMT1 (IC_50_ = 3.0 µM) using an enhanced formation of S-adenosyl-L–homocysteine (C_14_H_20_N_6_O_5_S) (SAH, a potent inhibitor of DNA methylation) during catecholamine-*O*-methyltransferase (COMT)-mediated O-methylation. Furthermore, CA could inhibit the methylation of the promoter region of the RAR-β gene using cultured MCF-7 and MAD-MB-231 human breast cancer cells [45]. CA has demonstrated its importance as an effective inhibitor of 5-lipooxygenase (5-LO) and it has proven its capacity to diminish NF-κB, Interleukin-6 (IL-6), and IL-1β levels during inflammatory processes, especially global cerebral ischemia-reperfusion injuries in rats [46]. Furthermore, in the study by Jung et al., CA radically inhibited the action of STAT3, which triggers the action of HIF-1α. It strongly inhibited STAT3 and suppressed tumor angiogenesis by blocking the signal transducer and activator of transcription 3 (STAT3) activity, as well as the vascular endothelial growth factor (VEGF) and hypoxia-inducible factor-1α (HIF-1α) expression [47]. Moreover, CA significantly suppressed CT-26 colon cancer cell-induced lung metastasis through the inhibition of ERK phosphorylation. CA also markedly repressed mitogen-activated MEK1 and TOPK functions and directly bound with MEK1 or TOPK. CA blocked TPA-, EGF-, and H-Ras-induced ERK phosphorylation, AP-1, and NF-κB transactivation, and the neoplastic transformation of JB6 P+ cells [48].

Regarding skin cancer, Yang et al. analyzed the molecular mechanisms by which CA could exert chemopreventive action against the EGF-induced neoplastic transformation of HaCaT cells and SUV-mediated skin carcinogenesis [49]. These researchers found that CA effectively inhibits colony formation and suppresses tumor incidence and volume in both in vivo and in vitro models. The CA-treated mouse skin cancer xenograft model showed a reduction in MAPK phosphorylation. Additionally, CA interacted with ERK2 directly in the Q105, D106, and M108 amino acid residues, directly interfered with ERK1/2, and suppressed ERK1/2 activities in vitro [49]. In lung cancer, CA phenethyl ester, a CA derivative representing a natural phenolic compound that is widely abundant in plants and propolis extract, was found to exhibit high chemopreventive effects against A549 lung adenocarcinoma cells. It was highly influential in suppressing TGF-β-promoted cell motility and transforming the growth factor-β (TGF-β)-induced activation of Akt (protein kinase β), and in blocking the phosphatidyl inositol 3-kinase (PI3K)/Akt pathway.

In prostate cancer, evidence showed that the CA phenethyl ester can block NF-κB activation in prostate cancer-3 (PC-3) cells by inhibiting the ability of the Tumor necrosis factor-α (TNF-α) and Paclitaxel to activate NF-κB; however, this action is also linked to lower cellular levels of apoptosis-inhibiting proteins (cIAP1, cIAP-2, and XIAP), and the downregulation of elevated levels of spontaneous apoptosis and cIAP-1 expression [33].

**Figure 3:**
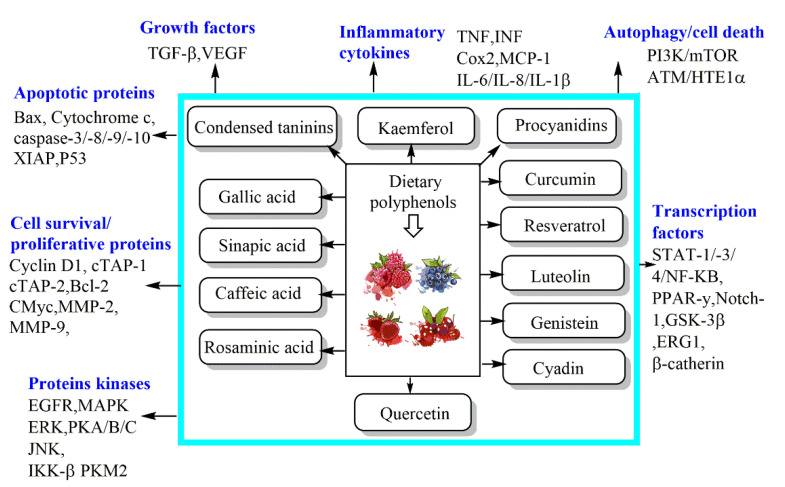
Mechanisms of dietary phenolic bioactive compounds against cancer cells.

Similarly, CA phenethyl ester exerted a strong anticancer effect against androgen-independent prostate cancer cells. It induced a protein abundance of p21Cip1, p27Kip1, ATF4, cyclin E, p53, TRIB3, phospho-p53 (Ser6, Ser33, Ser46, Ser392), phospho-p38 MAPK Thr180/Tyr182, Chk1, Chk2, phospho-ATM S1981, phospho-ATR S428, and phospho-p90RSK Ser380; however, it markedly decreased the protein abundance of Skp2, Cdk2, Cdk4, Cdk7, Rb, phospho-Rb S807/811, cyclin A, cyclin D1, cyclin H, E2F1, c-Myc, SGK, phospho-p70S6kinase T421/S424, phospho-mTOR Ser2481, and phospho-GSK3α Ser21. This investigation established that CA phenethyl ester induces cell cycle arrest and growth inhibition in castration-resistant prostate cancer cells through the regulation of Skp2, p53, p21Cip1, and p27Kip1 (Figure 3) [34].

Due to the high resistance of melanoma to conventional chemotherapy, several investigations have been undertaken that are aimed at new therapeutic strategies and the development of co-adjuvants. Using A2058 human melanoma cells, CA phenethyl ester was proposed to inhibit a ROS-induced DNA strand break [35]. Results revealed that the administration of CA phenethyl ester (10 mg/kg/day) causes intracellular glutathione (GSH) depletion, triggers a five to seven fold increase in B16-F0 cell apoptosis, and inhibits tumor size growth [36]. Moreover, CA exerted cell toxicity at concentrations above 0.35 mM and suppressed (in vitro) melanin production and casein kinase 2 (CK2)-induced tyrosinase phosphorylation in a dose-dependent manner [37]. In their study, Wadhwa et al. found that CA phenethyl ester exerts strong antitumor and antimetastatic potential, particularly when complexed with gamma-cyclodextrin (γCD). As shown via molecular docking analysis, this compound can disrupt mortalin-p53 complexes [38]. Furthermore, the study’s findings revealed that the disruption of the CA phenethyl ester-induced mortal-p53 complexes leads to the nuclear translocation and activation of p53, thus causing cancer cell growth arrest. Researchers also found that CA phenethyl ester is cytotoxic to several cancer cells (IC_50_ varies from 5 to 80 µM) when complexed with γCD [39].

To find new treatments for osteosarcoma, Pagnan et al. explored the anticancer effect of CA phenethyl ester, and its precursor CA, on murine osteosarcoma UMR-106. CA phenethyl ester (IC_25_ = 1.3 μM/IC_50_ = 2.7 μM) was found to diminish mitochondrial ROS production, cell migration, and cell viability when compared with CA alone (IC_25_ = 91.0 μM/IC_50_ = 120.0 μM) [40]. CA phenethyl ester showed greater selectivity towards tumor cells, thus protecting normal cells. These results illustrate the potential anticancer properties of CA phenethyl ester, and they highlight ways in which incorporating a phenethyl ester could lead to improved drug performance over its CA predecessor [40].

#### 4.1.3. Rosmarinic Acid

Rosmarinic acid (RA), (2*R*)-3-(3,4-Dihydroxyphenyl)-2-{[(*E*)-3-(3,4-dihydroxyphenyl)prop-2-enoyl]oxy}propanoic acid, one of the esters of caffeic acid, is a secondary metabolite of plants and is mainly found in plants belonging to the Lamiaceae family, such as *Rosmarinus officinalis* (rosemary) [111]. To explore the anticancer effects of RA, Luo and collaborators conducted a study using human oral cancer cells. They recorded the inhibition of cell proliferation, induction of apoptosis, cell cycle arrest at the G_2_/M phase, and the negative effect of cancer cell migration potential, in a concentration-dependent manner, while oxidative stress was being promoted in the endoplasmic reticulum [52]. Moreover, RA reduced levels of dextran sulphate sodium in (DSS)-induced colitis-associated colon cancer by triggering TLR4/NF-κB/STAT3 activation in the mouse model. RA dramatically improved the colitis severity, tumor incidence, colorectal adenoma progression, anti-apoptotic factors, and inflammatory markers [53].

To study the anticancer effects of RA against prostate tumors, Jang et al. conducted a study using prostate cancer cell lines [54]. The data obtained revealed that AR inhibits colony and spheroid formation and decreases cell proliferation in OC3 and DU145 cell lines. Additionally, RA treatment effectively suppressed a histone deacetylase enzyme that regulated apoptotic pathway-related mitochondrial intrinsic gene expression, including Bcl-2, Bax, caspase-3, and poly (ADP-ribose) polymerase-1 (PARP-1), through the enhancement of p53 levels. Furthermore, RA induced early and late apoptosis by downregulating the proliferating cell nuclear antigen, cyclin D1, and cyclin E1, and increasing p21 expression. Nevertheless, further studies are needed to assess AR-mediated cytotoxicity in normal cells [54]. Another study by Chao et al. investigated the RA’s anticancer effects on hepatocellular carcinoma [55]. Rosmarinic acid is among the significant polyphenolic components of the ethyl acetate fractionated extract (EAFE) of *Glechoma hederacea* L., which was found to be effective in inhibiting HepG2 cell proliferation, inducing apoptosis, and it also triggered cell death and arrest at the S phase. The apoptogenic activity of EAFE involves Ca^2+^ accumulation, ROS induction, MMP disruption, caspase 3, nine activations, an increased Bax/Bcl-2 ratio, and glutathione depletion.

In the same context, RA markedly suppressed SMMC-7721 cell proliferation and promoted G_1_ arrest and apoptosis in a dose-dependent manner. Additionally, RA appears to prevent cell invasion by controlling the epithelial–mesenchymal transition. RA treatment significantly inhibited the PI3K/AKT/mTOR signaling pathway. The findings above provide a basis for considering RA as an anti-cancer drug [56]. Concerning malignant bone tumors, the results obtained by Ma et al. indicated an anticancer property of RA in U2OS and MG63 osteosarcoma cells by suppressing DJ-1 expression via regulation of the PTEN/PI3K/Akt signaling pathway [57]. RA induced apoptosis by upregulating caspase-8, caspase-9, and caspase-3 cleavage rates, thus enhancing the Bax/Bcl-2 ratio, triggering ROS generation, and reducing the matrix metalloproteinase (MMP); therefore, a biological target of RA in osteosarcoma cells could be DJ-1 [57]. The anticancer activity of RA to prevent breast cancer has also been tested. In the study carried out by Messeha et al., the results revealed significant dose- and time-dependent antiproliferative and cytotoxic effects in the cell lines that were tested [58]. Using MDA-MB-231 cells, arrested AR cells in the G_0_/G_1_ phase, induced apoptosis that was related to cell cycle arrest, and differential alterations, many genes were involved in apoptosis expression [58].

In the MDA-MB-468 cell line, RA caused the significant transcriptional activation of TNF, growth arrest, DNA-damage-inducible 45 alpha (GADD45A), and BNIP3. In contrast, RA markedly upregulated the mRNA expression of harakiri (HRK), tumor necrosis factor receptor superfamily 25 (TNFRSF25), and BCL-2 interacting protein 3 (BNIP3) in MDA-MB-231 cells. In addition, RA suppressed TNF superfamily 11B receptor (TNFRSF11B) expression in MDA-MB-231 cells. Evidence indicates that RA may be a promising candidate for triple-negative breast cancer strategies, especially in MDA-MB-468 cells [58]. Similarly, using mouse models of breast cancer, RA exhibited chemopreventive benefits and antitumor effects alone and in combination with Paclitaxel. AR markedly repressed VEGF, NF-κB, and TNF-α expression and restored Bcl-2, p53, caspase-3, and Bax levels, thus causing apoptosis. Furthermore, RA and/or Paclitaxel markedly repressed tumor growth with increasing p53 and caspase-3 apoptotic marker levels and repressed the Bcl2/Bax ratio in mice with Ehrlich solid tumors [59].

To evaluate the anti-cancer effects in melanoma cells, Huang et al. reported that RA inhibits the invasion, proliferation, and migration of human melanoma A375 cells, promotes apoptosis, and increases theCisplatin sensitivity of melanoma cells by inhibiting the ADAM17/EGFR/AKT/GSK3β pathway [60]. Recently, Zhou et al. reported that RA restricts Gli1 nuclear translocation and promotes proteasome-mediated Gli1 degradation. RA resulted in G_1_/S cell cycle arrest and apoptosis in pancreatic ductal adenocarcinoma cells and inhibited the invasion and migration of these cells via MMP-9 and E-cadherin. These results strongly support RA as a beneficial therapeutic agent for pancreatic ductal adenocarcinoma cells [61]. In addition, RA treatment downregulated the oncogenic transcription factor forkhead box M1 (FOXM1) and targeted genes involved in tumor growth and abnormal cell proliferation. Furthermore, a combination of Cisplatin and RA methyl ester reversed Cisplatin resistance by potentially inhibiting FOXM1 [62] in ovarian cancer cells.

Moreover, RA dose-dependently suppressed non-small cell lung cancer (NSCLC) growth and cell colony formation, induced G_1_ phase cell cycle arrest and apoptosis, and increased the sensitivity of the Cisplatin-resistant cell line. The molecular mechanism of RA involved the inhibition of NSCLC cell growth, cell cycle arrest, and the induction of apoptosis through the activation of MAPK and suppression of P-gp and MDR1 expression, subsequently resulting in higher expression levels of p21 and p53 [63]. Interestingly, RA combined with anti-MUC1 showed stronger anticancer effects than monotherapy. Combined treatment significantly suppressed fucose, Tn, sialyl T, and cancer-related sugar antigen expression, as well as the mRNA expression of enzymes that are involved in their formation: ppGalNAcT2, C1GalT1, ST6GalNAcT2, ST3GalT1, and FUT4. Furthermore, C1GalT1 was suppressed at the protein level. Gal-3, which is a factor that is likely to be involved in metastasis, was effectively repressed at the mRNA level by administering RA and anti-MUC1. Bax protein and pro-apoptotic Bad mRNA were induced to a significant extent, and the expression of anti-apoptotic Bcl-2 mRNA was suppressed by this treatment [64].

#### 4.1.4. Sinapic Acid

Sinapic acid (SA), (2*E*)-3-(4-Hydroxy-3,5-dimethoxyphenyl)prop-2-enoic acid, can be found in a wide variety of fruits and vegetables, and is derived from hydroxycinnamic acid. To assess the role of SA as an anticancer agent for the prevention of prostate cancer, Eroğlu et al. have shown that SA has the potential to become a promising molecule that works against prostate cancer cells [65]. In LNCaP cells, BAX, caspase-3, caspase-7, and CYCS expressions markedly increased; however, reduced expressions of CDH2, MMP-2, and MMP-9 were observed in AS therapy. In PC-3 cells, treatment with AS enhanced BAX, caspase-3, caspase-8, CYCS, FAS, TIMP-1, and CDH1 expression, but significantly reduced the expression of MMP-9. The IC_50_ value of SA in both PC-3 and LNCaP cells was determined at 1000 μM for 72 h [65]. SA provided substantial protection against lipopolysaccharide (LPS)/D-galactosamine (D-GalN)-induced acute liver failure (ALF) in rats via the downregulation of NF-κB and upregulation of Nrf2/heme oxygenase (HO)-1 pathways [66]. SA is a histone deacetylase (HDAC) inhibitor that exerts its antitumor activity through apoptosis activation and autophagy induction, cell cycle arrest, enhanced ROS release leading to oxidative stress, the inhibition of angiogenesis, and induction of mitotic cell death in cancer cells [67]. Moreover, SA suppressed pancreatic cancer proliferation, migration, and invasion through the downregulation of the AKT/Gsk-3β signaling pathway [68].

Recently, Hu et al. examined the possible role of SA in combating lung cancer in vitro and in vivo [69]. The in vitro model showed that SA exhibits significant cytotoxic potential against human lung cancer, and it exhibited potential cytotoxic and apoptotic activity by increasing ROS levels, caspase-3, and caspase-9 activity. In the in vivo model, SA improved B[a]P-mediated lung cancer exposure in Swiss albino mice by reducing IgG and IgM levels, the number of leukocytes, assays of neutrophil function, soluble immune complexes, tumor markers (AHH, LDH, GGT, 5’NT, and CEA), pro-inflammatory cytokines, and by improving the activity index, phagocytic index, and the enzymes involved with antioxidant defense [69]. In addition, the effect of SA on the activation of apoptosis in the human laryngeal carcinoma cell line (HEp-2) was investigated in vitro by Janakiraman et al. [70]. As evidenced by the dichlorofluorescein diacetate (DCFH-DA) fluorometric method, SA-treated HEp-2 cells showed increased ROS levels (108.32 ± 7.32) compared with untreated cells (76.41 ± 7.09) for 48 h. The results also revealed a decrease in mitochondrial membrane potential in cancer cells with enhanced fluorescence intensity (134.78 ± 9.76) versus treated cells (123.76 ± 7.81), as shown in the fluorescence assay. The IC_50_ value of SA was 125.23 μM/mL after 24 h and 117.81 µM/mL after 48 h. SA promoted cell apoptosis with associated cell viability loss, modulated ROS, and arrested cell cycles by triggering G_0_/G_1_ phase arrest for HEp-2 cells.

In another study, Balaji mice injected with SA highlighted the chemopreventive properties of SA against DMH-induced colon carcinogenesis in vivo. The findings showed that SA showed a stronger action at a dose of 40 mg/kg body weight, which was measured by the increase in antioxidant defense (superoxide dismutase (SOD), catalase (CAT), and glutathione peroxidase (GPx)), a reduction in tumor incidence, and the modulation of LPO markers, including phase I (CYP450 and CYP2E1) and phase II Glutathione-S-transferase (GST), DT-Diaphorase (DTD), and UDP-Glucuronyl transferase (UDP-GT) detoxification enzymes [71]. Furthermore, the combination of SA and Cisplatin suppressed hepatocellular carcinoma cell migration and proliferation, as evidenced by the MTT assay, and it promoted autophagy and apoptosis, thus providing anti-hepatocellular carcinoma action [72].

### 4.2. Curcuminoids

#### Curcumin

The polyphenolic phytochemical Curcumin ((1*E*,6*E*)1,7-Bis(4-hydroxy-3-methoxyphenyl)hepta-1,6-diene-3,5-dione), extracted from *Curcuma longa*, is a powerful anti-proliferative, anti-angiogenic, anti-metastatic, pro-apoptotic, and antioxidant promoter of anticancer activities in several types of cancer cell lines [112]. Masuelli et al. explored the possible anticancer effects of Curcumin against human and mouse MM cell lines. Curcumin was found to induce DNA damage, suppress MM cell survival in a dose- and time-dependent manner, and increase ROS production [73]. It also activated apoptosis inductions via increased p53 expression, PARP-1 cleavage, caspase-9 activation, an increased Bax/Bcl-2 ratio, and cell cycle arrest. Curcumin treatment promoted ERK1/2 and p38 MAPK phosphorylation, blocked p54 JNK and AKT phosphorylation, enhanced c-Jun expression and phosphorylation, and prevented NF-κB nuclear translocation [73].

Chronic inflammation is one of the leading underlying causes of several types of cancer. In this sense, it was found that the activity of IκB kinase is reduced by curcumin, thereby inhibiting the degradation of IκBα and resulting in the blockage of NF-κB subunit nuclear translocation. Additionally, Curcumin can block the shift of subunit NF-κB to the nucleus and attenuate the expression of other pro-inflammatory genes, which are usually activated in cancer [113]. Moreover, it regulated the NF-κβ-dependent signaling pathways to induce apoptosis. Curcumin has also been documented to suppress p38 and JNK activation that is induced by TNF-α or IL-1β, thus leading to the potential suppression of IκBα degradation in HT29 cells [74].

Using head and neck squamous cell carcinoma cancer, Curcumin repressed inflammatory cytokines such as TNF-α, IKKβ kinase, IL-6, and IL-8. Additionally, Curcumin can suppress protein kinase activity, including PKA, PhK, mTOR, and MAPK, which are critical for regulating cell proliferation, growth, survival, and death [90]. In human glioblastoma, Curcumin has the potential to modulate critical pathways, including apoptosis, cell proliferation, cell cycle arrest, autophagy, tumor cell motility, and oxidative stress. The mechanisms of action involved in the anticancer effect of Curcumin in human glioblastoma include modulation of the Rb, p53, MAPK, P13K/Akt, JAK/STAT, Shh, and NF-κB pathways [75]. Similarly, curcumin reinforced nimustine hydrochloride’s (UNA) anticancer effect by deleting IκB, p65, and p50 phosphorylation and reducing the expression of cyclooxygenase-2 (COX-2) [76]. In addition, Curcumin’s antiproliferative activity could be mediated by the downregulation of cyclin D1, since NF-κB regulates the cyclin D1 promoter [77].

In the survey undertaken by Tzuu-Yuan et al., using GBM 8401 cells, Curcumin upregulated the inhibition of transcription factor NF-κB in a concentration-dependent manner [78]. It induced apoptosis through a caspase and mitochondrial-dependent pathway. Similarly, Curcumin promoted gefitinib’s antitumor activity in the xenografted NSCLC cell lines and mouse model via suppression of NSCLC proliferation, EGFR phosphorylation, EGFR ubiquitination, and apoptosis induction. In addition, Curcumin mitigated gefitinib-induced gastrointestinal side effects by altering p38 activation [79].

In lung adenocarcinoma cells, by suppressing the expression of ERK1/2 activities, as well as EGFR, and COX-2, Curcumin exhibited pro-apoptotic activity, which is consistent with high apoptosis and the reduced survival of pulmonary adenocarcinoma cells [91]. Similarly, in A549 human lung cancer cell lines, Curcumin downregulated NF-κB activity and acted on the JAK2/STAT3 signaling pathway by suppressing JAK2 activity; thus, Curcumin is a successful therapeutic drug for treating lung cancer [92]. Interestingly, a new catanionic lipid nanosystem (CLN) incorporating Curcumin was found to possess enhanced cytotoxicity in Lewis lung cancer cells, inducing cell cycle arrest, pro-apoptotic, antiproliferative, and anti-invasive effects. This novel nanosystem triggered Lewis lung cancer cell apoptosis with Curcumin via the cellular target PI3K/Akt/FoxO1/Bim [93].

In ML-1a human myeloid cells, researchers discovered that Curcumin represses TNF-α-induced nuclear translocation and NF-κB DNA binding by suppressing IκBα phosphorylation and degradation [94]. There is no cure for chronic lymphocytic leukemia (CLL) with current chemotherapy treatments; however, Curcumin was found to induce apoptosis in B-cell chronic lymphocytic leukemia (B-CLL) via the upregulation of the proapoptotic protein BIM, and the downregulation of the AKT, STAT3, and XIAP (X-linked inhibitor of apoptosis) proteins and NF-κB [95]. Using colony formation and MTT methods, Li et al. demonstrated that Curcumin exhibits anticancer activity against leukemic cell lines [96]. This compound suppressed clonogenicity and cell proliferation and arrested the cell cycle at the G_2_/M phase in a time- and dose-dependent manner through the inhibition of Wilms tumor protein 1 (WT1) [96].

The second most common hematological malignancy, multiple myeloma, may be incurable; however, in a recent paper, Curcumin was found to potently block MM cell proliferation by promoting apoptosis in a time- and concentration-dependent manner by blocking EZH2 expression in RPMI8226 and U266 cell lines. Curcumin enhanced miR-101, and after that, lower EZH2 expression was observed. Conversely, the expression of EZH2 resulted in a softer expression of miR-101. These results indicated Curcumin’s impact and mode of action on multiple myeloma via the EZH2-miR-101 regulating feedback circuit [96]. In breast cancer, it is essential to note that recent research has reported Curcumin’s potential to effectively target many breast cancer-related signaling pathways, including Wnt/β-Catenin, Hedgehog, Notch, and PI3K/mTOR, as well as JAK-STAT [81]. In addition, this phytochemical can suppress cell growth in breast cancer cells by epigenetic modifications and DNA methylation mechanisms [80]. On the other hand, a mixture of Curcumin and arabinogalactan markedly reduced the growth of breast cancer cells with no appreciable effect on normal cells. The combined treatment enhanced cell apoptosis by affecting the membrane potential, elevating the ROS level, and reducing glutathione. Furthermore, the combination of Curcumin and arabinogalactan inhibited breast tumor progression through the overexpression of p53 levels in mice [82].

Additionally, Curcumin reduced cell proliferation in MCF-7 breast cancer cells by arresting cells in the G_1_ phase. This cell cycle arrest is mediated by enhancing cyclin E proteaosomal degradation and increasing the upregulation of CDK inhibitors p21, p53, and p27 [83]. In human mammary epithelial carcinoma cells, Curcumin-induced apoptosis via a selective increase in G-phase p53 expression (2) generated cytochrome c from mitochondria, downregulated cyclin D1 expression, and blocked the Cdk4/Cdk6 association [84]. Furthermore, Curcumin showed anticancer effects by inhibiting invasion and inducing apoptosis via ROS production, thus decreasing MMP-2 and Bcl-2 activity, and increasing Bax and caspase-3 activity in H-ras-transformed MCF10A human mammary epithelial cells [85]. Garcea et al. showed in their investigation that Curcumin administration causes a downregulation of M(1)G protein levels in colorectal cancer, whereas COX-2 protein levels remain unaltered in malignant colorectal tissue [86]. It also effectively suppressed the growth of the HT-29 cell line and DMH (1,2-dimethylhydrazine)-induced colorectal carcinogenesis in rats by inhibiting the signal transduction pathway PPARγ [87]. Furthermore, Curcumin repressed (COX-2) the pre-RNA processing factor 4B (Prp4B) and p53 expression [88].

In liver cancer, findings showed that Curcumin improves stability and the cytoprotective effect against *tert*-butyl hydroperoxide (*t*-BHP)-induced HepG2 cell death, and it promotes the nuclear transport of transcription factor Nrf-2, which controls the antioxidant signaling pathway [98]. Curcumin also targets the Notch-1 signaling pathway. Indeed, notch-1 signaling was damaged by Curcumin in the intracellular Notch domain in HEP3B, SK-Hep-1, and SNU449 cell lines. It also showed a protective effect against diethylnitrosamine (DENA)-induced hyperplasia and HCC in rodents by reducing p21-Ras, p53, and NF-κB expression [97].

### 4.3. Flavonoids

#### Quercetin

Quercetin (3,3′,4′,5,7-Pentahydroxyflavone) is the main component of the flavonoid subclass, flavonols. It is found in numerous fruits and vegetables, and it constitutes one of the most common flavonols in the Western diet. This phytochemical plays a pivotal role in cancer prevention [114]. Within this context, Lei et al. explored the therapeutic effect of Quercetin combined with irinotecan/SN-38 on the human gastric cancer cell line AGS [99]. Consequently, Quercetin decreased cell viability in a concentration-dependent manner. At a dose of 50 µM, it inhibited 50% of tumor growth. Combined with SN-38, it improved apoptosis, enhanced the anti-proliferation action, and synergistically modulated GSK-3β/β-catenin signaling with SN-38. Quercetin, in combination with irinotecan (10 mg/kg once a week), or alone (three times a week), mediated a substantial decrease in tumor size by day 28, it downregulated tumors VEGF-R and VEGF-A, and decreased COX-2 gene expression [99].

Jia et al. indicated that Quercetin could block breast cancer progression by preventing the cell motility and glycolysis mediated by the Akt-mTOR pathway; thus, it may be a promising candidate for the treatment of breast cancer [100]. Quercetin induces autophagy through Akt-mTOR pathway inactivation. Moreover, by applying IGF-1, an Akt-mTOR pathway inducer, and 3-MA, an autophagy inhibitor, these authors showed that Quercetin exhibits an effective suppressive effect on glycolysis and cell motility through the activation of autophagy, which is mediated by the Akt-mTOR pathway. In addition, Quercetin downregulated MMP-2 and MMP-9, VEGF suppressed glucose uptake and lactate production, as well as suppressing PKM2, LDHA, and GLUT1 expression. Furthermore, Quercetin repressed the progression and metastasis of breast cancer in mice that were injected with MCF-7 cells. It also downregulated PKM2, p-AKT, and VEGF levels in tumor tissue [100].

Furthermore, Quercetin, at a physiologically relevant concentration (0–10 μM, 4-day incubation), dose-dependently suppressed the growth of breast cancer cell lines SK-Br3 and MDA-MB-453. Although a low dose of Quercetin exhibited a modest cytotoxic effect, the primary mechanism of Quercetin’s antiproliferative effect was cell cycle arrest in the G_1_ phase. This event occurred via the downregulation of cyclin-dependent kinase 1 (CDK1) and cyclin B1, which are critical ingredients for G_2_/M cell cycle progression, and via the activation of phosphorylation for the retinoblastoma tumor suppressor protein, pRb [102]. Moreover, Quercetin reduced cell proliferation in a concentration-dependent manner (0.6 to 300 μΜ) and inhibited five glycolysis pathway molecules that are related to ovarian and breast cancer cell lines, specifically GLUT1, HKII, PFKFB3, PDHK1, and LDH [101].

To show the beneficial effects of Quercetin against prostate cancer, Erdogan and colleagues showed that this substance, at a dose of 40 Μm, upregulates Bax, Bcl-2, p21Cip1, p27Kip1, and Cyt c, caspase 3, caspase 8, and p53. It also blocked cell survival in a dose- and time-dependent manner. This polyphenol was also found to enhance midkine’s apoptotic effect, increase caspase 3, and decrease survivin gene expression. It stimulated cell cycle arrest in the G_1_ phase and reduced S phase cells. Similarly, Quercetin downregulated PI3K, Akt, ERK1/2, p38, NF-κB, and survivin protein phosphorylation, and upregulated PTEN expression. In combination with midkine, Quercetin exhibited a stronger action on ERK1/2, p38, NFκB, and survivin [106]. In contrast, at a dose of 100 and 150 μM (added every 24 h for 72 h), Quercetin suppressed proliferation in human prostate adenocarcinoma cell lines (PC-3) by 83% and 64.17%. It regulated the gene expression associated with DNA repair, tumor invasion and matrix degradation, apoptosis, cell cycle arrest, glycolysis, and metabolism, without cytotoxicity [105].

To find new alternatives to treat primary effusion lymphoma (PEL), Granato et al. demonstrated that Quercetin suppresses the cytokine release of both IL-6 and IL-10, thus leading to PEL cell death. At the same time, Quercetin inhibited the PI3K/AKT/mTOR and STAT3 pathways in PEL cells, thereby decreasing the level of cell survival proteins, including c-FLIP, cyclin D1, and cMyc [104]. Similarly, Sturza et al. evaluated the beneficial activities of Quercetin on the bioenergetic profile of the B164A5 murine melanoma cell line [103]. Results showed that Quercetin might represent an alternative strategy to treat cancer cells by modulating mitochondrial and glycolytic pathways to produce ATP. Moreover, Quercetin could reduce the extracellular acidification rate (ECAR) and oxygen consumption rate (OCR) by 50, 100, and 150 µM after a 48 h treatment [103].

## 5. Dietary Phenolic Compounds as Anticancer Drugs: Clinical Trials

### 5.1. Curcuminoids

#### Curcumin

Curcumin was reported in the literature as a chemotherapeutic, chemopreventive, and chemosensitizer agent [115] due to its various anticancer properties, as shown in the study by Chapman et al. [116]. With this in mind, Dhillon et al. assessed the oral administration of 8 g/day of curcumin to patients with pancreatic cancer [117]. The antitumor effects of curcumin were observed in two patients; one showed progress where curcumin treatment reduced the tumor by 73% without toxicity at 8 g/day for up to 18 months, however, the limited bioavailability of curcumin likely attenuated the activity. Additionally, Sung et al. tested curcumin on multiple myeloma (MM) patients in phase I/II clinical trials [118]. The study revealed that all doses (2, 4, 8, and 12 g/day) showed no side effects and inhibited the activation of NF-κB, COX-2, and STAT3 in the peripheral blood mononuclear cells (PBMC) that were isolated from MM patients. Interestingly, Cruz et al. reported that the combined treatment of Curcumin and Quercetin (480 mg and 20 mg, respectively, thrice a day, for six months) exerted adenoma regression in five patients with familial adenomatous polyposis (FAP) [119]. This treatment significantly reduced the number and size of ileal and rectal adenomas, compared with the baseline (60.4% and 50.9%, respectively), without toxicity. Other studies demonstrated that a daily dose of curcumin (3.6 g in capsule form) improves the general health of colorectal cancer patients by increasing p53 molecule expression in tumor cells, and consequently, inducing tumor cell apoptosis [86,120].

### 5.2. Stilbenes

#### Resveratrol

Resveratrol, also called *trans*-3,5,4′-Trihydroxystilbene and 5-[(*E*)-2-(4-Hydroxyphenyl)ethen-1-yl]benzene-1,3-diol, is a remarkable bioactive compound. In recent decades, several clinical studies have been conducted to provide clear data on the pharmacokinetics, safety, and effectiveness of Resveratrol to prove its therapeutic efficacy against different cancers; however, most of these investigations focus primarily on assessing Resveratrol’s bioavailability as a chemopreventive agent rather than its potential efficacy as a therapeutic agent for cancer patients. In this respect, a closed phase I clinical trial has shown the role of this molecule in the prevention of colon cancer [121]. Results demonstrated the potential effect of Resveratrol on the Wnt pathway, which is a pivotal pathway that is activated in more than 80% of colon cancers. Furthermore, in a phase I clinical trial, Resveratrol significantly reduced the levels of circulating cancer biomarkers, including Insulin-like growth factor 1 and Insulin-like growth factor-binding protein 3 [122]. In another phase I, double-blind, randomized clinical trial, Resveratrol increased the levels of cleaved caspase-3 in malignant hepatic tissue [123].

Generally, most clinical trials that are concerned with using this Stilbene against cancers in colorectal cancer patients note that Resveratrol could easily reach tumors located along the gastrointestinal tract at higher doses after oral administration. In their clinical study, Patel et al. showed that a concentration of 0.5 g Resveratrol effectively decreased the proliferation of colorectal cells [124]. On the other hand, ongoing clinical trials in phases I and II that either use Resveratrol alone or in combination with SRT501, demonstrated promising results in patients with colon and colorectal cancer [125]. After oral administration, the safety of micronized Resveratrol SRT501 has also been reported, and it showed a positive effect in colorectal cancer patients with hepatic metastases [125].

### 5.3. Flavonoids

#### 5.3.1. Isoflavones

##### Genistein

Genistein (4′,5,7-Trihydroxyisoflavone), 5,7-Dihydroxy-3-(4-hydroxyphenyl))-4*H*-1-benzopyran-4-one is a natural isoflavone that has been isolated from soybeans and is known as an antimetastatic agent. It inhibited tumor growth and metastasis in vivo and exhibited significant antiproliferative activity on several cancer cells in vitro [126,127,128]. Case-control studies showed that dietary soybean intake may reduce one’s cancer risk [129,130]. The clinical outcome proves the efficacy of Genistein as an antimetastatic agent for patients with prostate cancer. In a randomized, placebo-controlled, double-blind assay, Genistein significantly decreased serum prostate-specific antigen (PSA) levels in prostate cancer patients without affecting hormones [131]. Phase I and Phase II clinical trials have indicated that this soy isoflavone may reduce the risk of both hormone-dependent and hormone-independent cancers, including prostate, breast, gastric, non-small cell lung, and colon cancers [132]. The use of this molecule as a dietary supplement in the treatment of patients with stage II, III, or IV prostate cancer is under evaluation [129]. Additionally, the combinatory treatment of the soy isoflavone Genistein with Gemcitabine, a known cancer drug, is also being studied in women with stage IV breast cancer. A phase II, randomized, placebo-controlled trial, recruited 59 patients with urothelial bladder cancer, and showed that daily oral intake of Genistein (300 or 600 mg/d as the purified soy extract G-2535) for 14–21 days prior to surgery exhibits a bimodal effect on bladder cancer tissue, which is noticeable at the lowest concentration [133].

#### 5.3.2. Flavonols

##### Epigallocatechin-3-Gallate (EGCG)

Epigallocatechin-3-gallate (EGCG) is one of the phenolic compounds found in green tea, and it is considered a potent chemopreventive agent. It has been identified as an important constituent of polyphenol E, a standardized decaffeinated green tea catechin mixture that is currently used in numerous clinical trials [134,135]. To this end, EGCG is the subject of several investigations that aim to illustrate its chemosensitizing potential. It enhanced the therapeutic efficacy of the available chemotherapy treatments and reduced its associated adverse effects. In this respect, several clinical human trial-based studies in phases I and II have proven the critical role of EGCG in cancer prevention; however, further clinical trials are still needed to better understand the effectiveness of EGCG in cancer management.

The results of a study by McLarty et al., conducted on 26 men with positive prostate biopsies, showed that treatment with polyphenol E (800 mg EGCG for a total of 1.3 g of green tea) led to a significant reduction in serum levels of PSA, HGF, and VEGF, thus suggesting the efficacy of polyphenol E in the treatment or prevention of prostate cancer [136]. Similarly, Zhao et al. investigated the tolerability, safety, and efficacy of topical EGCG for radiation dermatitis in women with breast cancer undergoing radiotherapy [137]. These researchers indicated the good tolerance of EGCG following topical administration, although the maximum tolerated concentration was not reported. A phase I clinical trial with EGCG, in combination with standard chemoradiotherapy in unresectable stage III lung cancer, showed that oral administration of EGCG is feasible, safe, and effective [138]. Clinical phase II suggested a dose of 440 µmol/L [138].

## 6. Conclusions and Perspectives

At present, the use of foodstuffs in treating and preventing diseases, including cancer, is attracting attention. This is due to the presence of bioactive compounds such as polyphenols, among others, in our diet. These natural compounds are gaining popularity in cancer treatment due to their lower side effects, cost, and accessibility compared with conventional drugs. In this review, we have shown through published research that dietary phenolic compounds are an excellent source of natural anticancer substances, and they provide a range of preventive and therapeutic options against several types of cancer. These compounds could be used alone or in combination with other anticancer drugs. Certain phenolic compounds, such as Quercetin and Gallic acid, have well-known mechanisms of action. These molecules act specifically on the various checkpoints of cancerous cells; therefore, exploring these mechanisms of action could further improve therapeutic efficacy. However, further investigations that could involve human subjects and different pharmacokinetic parameters are required to ensure the safety of these compounds before they can be used as prescription drugs. In addition, the development of a standardized extract or dosage could also be studied in clinical trials. In summary, the polyphenols present in our food can be useful in complementary medicine for the prevention and treatment of different types of cancers due to their natural origin, safety, and low cost compared with cancer drugs. In our opinion, these molecules, that are contained in plants and used in our alimentary system, can be considered as functional foods or nutraceuticals. As a result of the different mechanisms demonstrated, phenolic compounds can prevent the appearance of certain cancers and reduce their progression towards later stages; however, further investigations need to be carried out on other cancers, as well as the risk factors that are associated with these cancers.

## Figures and Tables

**Table 1:** Dietary polyphenols that act as anticancer agents.

Dietary Polyphenols	Investigated Cell Lines	Key Results	Ref.
Gallic acid	HeLa (Cervical) and MDA-MB-231 (Breast) cancer cells (in vitro)	HeLa: IC_50_ = 4.1 µg/mL at 24 h MDA-MB231: IC_50_ = 80 µg/mL at 24 h ΔG of −5.4 kcal/mol to Bcl-B protein Ki = 110 μM	[24]
	Non-small cell lung carcinoma (NSCLC) cell line A549 (in vitro)	Induced cell cycle arrest/apoptosis by PI3K/Akt pathway Inhibited cell proliferation Upregulated p53	[25]
	A549-derived tumor xenograft model (in vivo)	Reduced tumor mass size (in vivo) Downregulated PCNA/p-Akt Upregulated caspase-3 in tumor tissues	
	Human liver cancer cell line (HepG2)	Induced anticancer action (58.90% toxicity) towards HepG2 cells at 1000 µg/mL	[26]
	CRL1790, SW480, and SW620 cell lines	CRL1790: IC_50_ = >100 µM SW480: IC_50_ = 22.3 µM SW620: IC_50_ = 11.8 µM Induced a cell cycle arrest Caused nucleolar stress Downregulated G-quadruplexes containing genes Stabilized G-quadruplexes in a cellular environment Induced DNA damage Binds to G4s present in 5′ETS/the promoter of CMYC	[27]
	Mouse xenograft model of CRC	Blocked tumor progression and stabilized G4 structures	
Gallic acid and its alkyl esters	Human prostate carcinoma DU145 cells	Showed growth inhibition and death Induced apoptotic death Caused caspase-9/caspase-3/PARP cleavages	[28]
		Inactivated cdc25A/cdc25C-cdc2 Induced cell cycle arrest Induced apoptosis Caused caspase-9/caspase-3/PARPs cleavage	[29]
	Human leukemia cell line HL-60	Potentially inhibited metastasis Induced apoptosis	[30]
Gallic acid	Oral squamous cell carcinoma (FaDu and Cal33) cell line	Promoted chemo-sensitization potential Triggered apoptosis via NRF2 inactivation	[31]
	Human promyelocytic leukemia (HL60RG) Mouse lymphoid neoplasm (P388- D1) Human epithelial carcinoma (HeLa) Rat hepatoma (dRLh-84) Human hepatoma (PLC/PRF/5) Human epidermoid carcinoma (KB)	HL60RG: IC_50_ = 5.4 µg/mL P388- D1: IC_50_ = 4.8 µg/mL HeLa: IC_50_ = 6.1 µg/mL dRLh-84: IC_50_ = 6.2 µg/mL PLC/PRF/5: IC_50_ = 6.6 µg/mL KB: IC_50_ = 13.2 µg/mL Endothelial cell/fibroblast: IC_50_ > 20 µg/mL	[32]
Caffeic acid and its derivative caffeic acid phenethyl ester:	A549 lung adenocarcinoma cells	Suppressed TGF-β-enhanced cell motility Suppressed TGF-β-induced Akt activation Blocked PI3K/Akt inhibitor	[33]
	Prostatic cancer-3 (PC-3) cells/DU145 cells	Blocked Paclitaxel and TNF-α to activate NF-κB Reduced cIAP-1/cIAP-2/XIAP Downregulated cIAP-1 expression	
	LNCaP 104-R1, DU-145, 22Rv1, and C4–2 CRPC cells	Induced cell cycle arrest Suppressed cell survival Induced growth inhibition Significantly reduced protein abundance of Skp2, Cdk2, Cdk4, Cdk7, Rb, phospho-Rb S807/811, cyclin A, cyclin D1, cyclin H, E2F1, c-Myc, SGK, phospho-p70S6kinase T421/S424, phospho-mTOR Ser2481, and phospho-GSK3α Ser21 Induced p21Cip1, p27Kip1, ATF4, cyclin E, p53, TRIB3, phospho-p53 (Ser6, Ser33, Ser46, Ser392), phospho-p38 MAPK Thr180/Tyr182, Chk1, Chk2, phospho-ATM S1981, phospho-ATR S428, and phospho-p90RSK Ser380 Decreased Skp2 and Akt1 protein expression	[34]
	Human melanoma A2058 cells	IC_50_ = 15 µM Suppressed ROS-induced DNA strand breakage	[35]
	Melanoma cell lines: B16- F0/B16F10/SK-MEL-28/SK-MEL-5/MeWo Skin B16-F0 melanoma tumor model in C57BL/6 mice	Depleted GSH Inhibited tumor size growth	[36]
	Murine B16 melanoma cell line	Exerted cellular toxicity at concentrations higher than 0.35 mM Inhibited melanin production Inhibited CK2-mediated phosphorylation of tyrosinase	[37]
	Molecular docking analysis	Disrupted mortalin-p53 complexes Exhibited down-regulation of mortalin Showed high efficacy in antitumor and anti-metastasis	[38]
	HT1080 cells in subcutaneous xenografts Cancer cells (SKOV3 and IMR32, HELA, A549)	IC_50_ ranged from 5 to 80 µM Induced disruption of mortalin-p53 Induced growth arrest Acted as a cytotoxic agent	[39]
	UMR-106 murine osteosarcoma cells	CAPE: IC_25_ = 1.3 μM/IC_50_ = 2.7 μM CA: IC_25_ = 91.0 μM/IC_50_ = 120.0 μM Decreased cell viabilityDecreased ROS/cell migration	[40]
Caffeic acid	Non-small-cell lung cancer (NSCLC) H1299 cells	Reduced cell proliferation Caused cell cycle arrest Increased apoptosis Increased caspase-3/caspase-9 Increased the PTX-induced activation of Bax/Bid downstream cleaved PARP Increased phosphorylation kinase1/2/c-Jun NH2-terminal protein kinase1/2	[41]
	H1299 xenografts	Exerted suppressive effect on tumor growth Showed no significant adverse effects	
	HeLa and ME-180 cancer cells	Exhibited pro-oxidant property Enhanced lipid peroxidation Enhanced ROS levels Altered mitochondrial membrane potential Increased apoptotic morphological changes	[42]
	Human peripheral lymphocytes	Induced pro-oxidant DNA breakage Generated oxidative stress	[43]
	Human hepatoma HepG2 cells	Induced cytotoxicity Induced apoptosis	[44]
	MCF-7 and MAD-MB-231 human breast cancer cells	IC_50_ = 3.0 µM Inhibited DNA methylation Increased SAH Inhibited methylation of RAR β gene	[45]
	Global cerebral ischemia-reperfusion injury in rats	Inhibited 5-LO Downregulated IL-6/IL-1β/NF-κB	[46]
	Human renal carcinoma and COS7 monkey kidney cell lines	Inhibited STAT3 Suppressed tumor angiogenesis Blocked STAT3/VEGF/HIF-1α	[47]
	CT-26 colon cancer cell-induced lung metastasis	Blocked ERKs/NF-κB/AP-1 Targeted MEK1/TOPK Inhibited TPA/EGF	[48]
	Human skin cancer cells and EGF-induced neoplastic transformation of HaCaT cells	Inhibited ERK1/2 Interacted directly with ERK2 at Q105/D106/M108 Inhibited colony formation	[49]
	Solar UV (SUV)-induced skin carcinogenesis mouse model	Suppressed tumor incidence Inhibited ERK1/2 Inhibited colony formation	
	ER+ (MCF-7) and ER− (MDA-MB-231) breast cancer cells	Suppressed ER+/ER− Limited the activities of antiestrogens Modified ER/cyclin D1/IGF-IR/pAkt Damaged cell-cycle development Decreased cellular proliferation	[50]
		Decreased IL-12/NF-κB Hindered TLR4 Decreased TRIF/TLR4/IRAK4 Triggered apoptosis	[51]
Rosmarinic acid	Human oral cancer cell line SCC-15	Inhibited cell proliferation Induced apoptosis Arrested cell cycle Negatively affected migratory potential of cancer cells Promoted endoplasmic reticulum oxidative stress Promoted the cleavage of pro-caspase-3	[52]
	AOM/DSS-induced CAC murine model	Suppressed NF-κB/STAT3 activation Suppressed colitis-associated tumorigenesis Abrogated human colon cancer progression Significantly modulated TLR4-mediated NF-κB and STAT3 activation	[53]
	OC3 and DU145 cell lines	Decreased cell proliferation Induced early and late-stage apoptosis Downregulated cyclin, D1, and cyclin E1 Upregulated p21 expression Inhibited a histone-deacetylase enzyme Modulated Bax/bcl-2/caspase-3/PARP1	[54]
	Hepatocellular carcinoma cell line HepG2 cells	Inhibited cell proliferation Caused S phase arrest Induced: ROS induction, Ca^2+^ 19 accumulation, glutathione depletion, MMP disruption, caspases 3, and 9 activation, and Bax/Bcl-2 ratio increment	[55]
	Hepatocellular carcinoma cell line SMMC-7721	Induced G_1_ arrest and apoptosis Inhibited the epithelial to mesenchymal transition Decreased tumor growth Inhibited PI3K/p-Akt/p-mTOR activation	[56]
	U2OS and MG63 osteosarcoma cells	Exerted anti-proliferation/pro-apoptotic effects Induced apoptosis Suppressed the migration and invasion Inhibited the expression levels of MMP-2/-9	[57]
	Triple-negative breast cancer (TNBC) cell lines	Caused cytotoxic effects Exhibited antiproliferative effects Induced cell cycle arrest Altered apoptosis-involved genes MDA-MB-231 cells: arrested cells at the G_0_/G_1_ phase MDA-MB-231 cells: upregulated mRNA expression HRK/TNFRSF25/BNIP3 MDA-MB-468 cell line: induced transcription activation of TNF/GADD45A/BNIP3 MDA-MB-231: repressed the expression of TNFRSF11B	[58]
	Ehrlich-induced mice mammary solid tumor model	Suppressed NF-κB/TNF-α/VEGF serum levels/VEGF receptors Triggered apoptosis Restored the levels of p53/Bcl-2/Bax/caspase-3 Suppressed tumor growth Increased apoptotic markers p53 and caspase-3 levels Suppressed the Bcl2/Bax ratio	[59]
	Human melanoma A375 cells	Inhibited invasion, proliferation, and migration Promoted apoptosis Increased Cisplatin sensitivity Inhibited ADAM17/EGFR/AKT/GSK3β axis	[60]
	Pancreatic ductal adenocarcinoma	Reduced malignancy Downregulated Gli 1 Facilitated proteasomal degradation Induced G_1_/S cell cycle arrest Induced apoptosis Regulated apoptotic genes’ expression: p21/p27, CDK2/Cyclin E/Bax/Bcl-2	[61]
Rosmarinic acid methyl ester, a derivative of rosmarinic acid	Ovarian cancer cells	Inhibited cell migration and invasion Decreased the mRNA expression of FOXM1 Reversed Cisplatin resistance	[62]
Rosmarinic acid	Non-small cell lung cancer	Inhibited cell proliferation Inhibited cell colony formation Induced G_1_ phase cell cycle arrest Induced apoptosis Reversed Cisplatin resistance Inhibited cell growth Arrested cell cycle Activated MAPK Inhibited the expression of P-gp and MDR1	[63]
Rosmarinic acid combined with anti-MUC1	AGS gastric cancer cells	Suppressed MUC1 mRNA Inhibited expression of Tn, T, sialyl Tn, sialyl T, and fucosylated sugar antigens Inhibited mRNA expression of ppGalNAcT2, C1GalT1, ST6GalNAcT2, ST3GalT1, and FUT4.	[64]
Sinapic acid	PC-3 and LNCaP cell lines	IC_50_ = 1000 μM for 72 h Suppressed cell invasion In PC-3 cells: increased BAX/Caspase-3/Caspase-8/CYCS/FAS/TIMP-1/CDH Decreased *MMP*-*9* In LNCaP cells: increased *BAX/*Caspase-3*/*Caspase-7*/CYCS* Decreased CDH2/*MMP*-*2*/*MMP*-*9*	[65]
	LPS/D-GalN-induced ALF in rats	Upregulated Nrf2/HO-1 Downregulated NF-κB	[66]
	Various types of cancer cells Osteosarcoma, breast, colorectal, prostate, lung cancer cells	Inhibited HDAC Activated cell cycle arrest Induced apoptosis and autophagy Inhibited angiogenesis Increased ROS generation Induced mitotic cell death	[67]
	Pancreatic cancer cells	Inhibited cell proliferation, migration, and invasion Downregulated the AKT/Gsk-3β signal pathway	[68]
	Human lung cancer cell (A549)	Showed potential cytotoxicity Induced apoptotic activity Increased ROS production and caspase activity (caspase-3 and caspase-9) in vitro	[69]
	B[a]P-stimulated lung cancer in Swiss albino mice	Ameliorated the exposure of B[a]P mediated lung cancer in vivoDeclined IgG and IgM level, leukocyte count, neutrophil function tests, soluble immune complex, lipid peroxidation, pro-inflammatory cytokines, tumor markers (AHH, LDH, GGT, 5’NT, and CEA) Enhanced phagocytic index, activity index, and antioxidant defense enzymes	
	Human laryngeal carcinoma cell line (HEp-2)	IC_50_ = 125.2 μM/mL for 24 h IC_50_ = 117.8 µM/mL for 48 h Enhanced apoptosis Induced loss of cell viability Increased ROS generation Arrested cell cycle	[70]
	1,2-Dimethylhydrazine (DMH)-induced rat colon carcinogenesis	Most effective at the dose of 40 mg/kg b.w Increased SOD, CAT, and GPx Reduced tumor incidence Modulated LPO markers	[71]
	Hepatoma Cells HepG2 and SMMC-7721	Inhibited cancer cell proliferation Induced cell apoptosis Activated autophagy	[72]
Curcumin	Mesothelioma (MM-F1 and MM-B1) cell lines	Induced apoptotic cell death Inhibited MM cells survival Induced DNA damage Upregulated Bax Downregulated Bcl-2 Stimulated ERK1/2/p38 MAPK Inhibited p54 JNK/AK Increased c-Jun expression Prevented NF-κB	[73]
	Human intestinal epithelial cell line HT29	Decreased binding of Shiga-like toxin-1B Suppressed TNFα or IL-1β-induced p38 and JNK activation	[74]
	Human Glioblastoma	Modulated cell proliferation, apoptosis, cell cycle arrest, autophagy, paraptosis, oxidative stress, and tumor cell motility Modulated Rb, p53, MAPK, P13K/Akt, JAK/STAT, Shh, and NF-κB pathways	[75]
	Human glioblastoma cell lines (U118MG, U87MG, and U251MG)	Enhanced the anticancer effect ACNU Suppressed the phosphorylation of IκB, p65, and p50 Decreased COX-2 expression	[76]
	NP-2 and NP-3 human malignant astrocytoma cell lines	Downregulated cyclin D1 Reduced NF-κB activity Inhibited cell proliferation Induced apoptosis	[77]
	Glioblastoma multiforme (GBM) 8401 Cells	Inhibited cell growth Induced apoptosis	[78]
	NSCLC cell lines	Inhibited cell proliferation Inhibited EGFR phosphorylation Induced EGFR degradation Induced apoptosis Modulated p38 activation	[79]
	Human breast cancer cells	Inhibited cell growth Modulated DNA methylation	[80]
	Breast cancer stem cells	Targeted numerous signaling pathways: Wnt/β-Catenin, Hedgehog/Notch/PI3K/mTOR/JAK-STAT	[81]
	Human breast cancer cells	Induced apoptosis Increased ROS level Changed mitochondrial membrane potential Reduced glutathione	[82]
	MCF-7 breast cancer cells	Reduced cell proliferation Induced cell cycle arrest at the G_1_ phase Stimulated the proteasomal degradation of cyclin E Up-regulated CDK inhibitors, p53, p21, and p27	[83]
	Human mammary epithelial carcinoma cells	Induced apoptosis Selectively increased p53 expression at the G_2_ phase Generated cytochrome c from mitochondria Downregulated the cyclin D1 expression and blocked Cdk4/Cdk6 association Inhibited phosphorylation and inactivation of retinoblastoma protein	[84]
	H-ras-transformed MCF10A human breast epithelial cells	Induced ROS production Downregulated the activity of MMP-2 and Bcl-2 Upregulated the activity of Bax and caspase-3	[85]
	Malignant colorectal tissue	Reduced M(1)G protein levels Did not affect COX-2 protein levels	[86]
	HT-29 cell line	Inhibited DMH (1,2-Dimethylhydrazine)-induced rat colorectal carcinogenesis Inhibited cell growth Suppressed PPARγ signal transduction pathway	[87]
	HT-29 cell line	Inhibited COX-2	[88]
	Human colorectal carcinoma (HCT-15)	Induced apoptosis Regulated Prp4 and p53	[89]
	Head and neck squamous cell carcinoma cancer	Suppressed TNF-α/IKKβ kinase/IL-6/IL-8 Suppressed the activity of PKA/PhK/Mtor/MAPKs	[90]
	Lung adenocarcinoma cells	Exhibited pro-apoptotic activity Reduced survival Downregulated COX-2 and EGFR activity Inhibited ERK1/2 activity	[91]
	Human lung cancer cell lines A549	Downregulated NF-κB Acted on the JAK2/STAT3 Inhibited JAK2 activity	[92]
	Lewis lung cancer (LLC) cells	Exhibited cytotoxicity Increased antiproliferative, proapoptotic, and anti-invasive activities Induced cell cycle arrest Caused apoptosis via the PI3K/Akt/FoxO1/Bim cellular target	[93]
	Human myeloid ML-1a cells	Suppressed TNF-α-induced nuclear translocation and DNA binding of NF-κB Suppressed IκBα phosphorylation and degradation	[94]
	B-cell chronic lymphocytic leukemia (CLL-B)	Induced apoptosis Downregulated STAT3, AKT, NF-κB, and XIAP Upregulated BIM	[95]
	K562 cell line	Caused cell cycle arrest at the G_2_/M phase Inhibited WT1 protein Inhibited cell proliferation Inhibited clonogenicity	[96]
	MM cell lines RPMI8226 and U266	Significantly inhibited cell proliferation Induced apoptosis Inhibited the expression of EZH2 Upregulated miR-101	[96]
	Hepatocellular carcinoma (HCC)	Targeted Notch-1 signaling pathway Damaged Notch-1 signaling within the Notch intracellular domain in the HEP3B, SK-Hep-1, and SNU449 cell lines Exhibited protection against DENA-induced hyperplasia Decreased the expression of p21-Ras, p53, and NF-κB	[97]
	1,1-Dimethylethyl hydroperoxide-induced death of HepG2 cells	Served as an Nrf2 activator Served as a cytoprotector against oxidative death	[98]
Quercetin	AGS human gastric cancer cell line	Reduced cell viability Combined with SN-38: improved the anti-proliferation effect and increased apoptosis Acted with SN-38 in the modulation of GSK-3β/β-catenin signaling	[99]
	BALB/c nude mice injected with AGS cells	Promoted a significant reduction in tumor size Reduced tumor VEGF-R and VEGF-A levels and protein levels Reduced COX-2 gene expression Decreased the TEM population	
	MCF-7 and MDA-MB-231 cell lines	IC_50_ = 30 Μm Decreased cell viability Increased autophagy Suppressed migration rate Reduced MMP-2, MMP-9, and VEGF Suppressed glucose uptake and lactate production Suppressed PKM2, LDHA, and GLUT1 expression Suppressed activation of AKT, mTOR, and p70-S6K	[100]
	Mice injected with MCF-7 cells	Inhibited tumor metastasis and progression Decreased VEGF, PKM2, and p-AKT levels	
	MCF-7, MDA-MB-231, HBL100, and BT549 breast cancer cells, and OVCAR5, TOV112D, OVCAR3, CAOV3 ovarian cancer cells	Blocked glycolysis Increased extracellular glucose Decreased lactate production Inhibited GLUT1, HKII, PFKFB3, PDHK1, and LDH Increased apoptosis	[101]
	MCF-7 cells	Increased apoptosis by 25%	
	HBL100 cells	Increased intracellular accumulation of glucose and promoted lactate depletion in the culture media	
	Breast cancer cell lines SK-Br3 and MDA-MB-453	Inhibited cell proliferation in a dose-dependent manner Downregulated cyclin B1 and CDK1 Induced phosphorylation of pRb	[102]
	B164A5 murine melanoma cell line	Decreased OCR and ECAR	[103]
	BC3, BCBL1, and BC1 PEL cell lines	Reduced cell survival and growth Increased G_1_ cell phase/PARP cleavage/nuclear fragmentation/condensation Inhibited PI3K/AKT/mTOR and STAT3 Promoted degradation of β-catenin	[104]
	Human prostate adenocarcinoma cell line (PC-3)	Reduced cell proliferation to 83% and 64% at 100 and 150 μM, respectively No cytotoxicity Modulated the expression of genes involved in DNA repair, matrix degradation and tumor invasion, angiogenesis, apoptosis, cell cycle, metabolism, and glycolysis	[105]
	Human prostate adenocarcinoma cell line (PC-3)	Inhibited survival Increased Cyt c, caspase-3, caspase-8, Bax, Bcl-2, p21_Cip1_, p27_Kip1_, and p53 Improved the apoptotic effect of MKsi Increased caspase-3 and decreased survivin gene expression Promoted cell cycle arrest in G_1_ phase cells Decreased cells in the S-phase Decreased the phosphorylation of PI3K, Akt, and ERK1/2 Decreased p38/NFκB Increased the PTEN expression Showed significant effect on ERK1/2, p38, and NF-κB, and survived when combined with MKsi	[106]
	HeLa (Cervical) and MDA-MB-231 (Breast) cancer cells (In vitro)	HeLa: IC_50_ = 99.9 µg/mL MDA-MB231: IC_50_ = 18.2 µg/mL at 24 h	[24]

## Data Availability

No new data were created or analyzed in this study. Data sharing is not applicable to this article.

## Abbreviations

**5-LO**	5-LipoOxygenase
**5’ ETS**	5’ External transcribed spacer
**5-FU**	5-Fluorouracil
**5’-NT**	5’-Nucleotidase
**Akt**	Protein Kinase B
**ADAM17**	Disintegrin and metalloprotease 17
**ATF4**	Activating transcription factor 4
**AHH**	Aryl hydrocarbon hydroxylase
**ATM**	Ataxia telangiectasia mutated
**ALF**	Acute Liver Failure
**AP-1**	Activator Protein-1
**BNIP3**	BCL-2-Interacting Protein 3
**BAX**	Bcl-2-associated X protein
**BCL-2**	B-cell leukemia/lymphoma-2
**CA**	Caffeic acid
**CEA**	Carcinoembryonic antigen
**Chk2**	Checkpoint kinase 2
**CAT**	Catalase
**CDK1**	Cyclin-Dependent Kinase 1
**CK2**	Casein Kinase 2
**CLL**	Chronic Lymphocytic Leukemia
**CLN**	Catanionic Lipid Nanosystem
**CDH-2**	Cadherin-2
**CYCS**	Cytochrome C, Somatic
**CMYC**	C-myc proto-oncogene protein
**COMT**	Catecholamine-O-Methyltransferase
**COX-2**	Cyclooxygenase-2
**CRC**	Colorectal Carcinoma
**DNMT**	DNA Methyltransferase
**DCFH-DA**	Dichlorofluorescein diacetate
**DSS**	Dextran Sulphate Sodium
**DTD**	DT-Diaphorase
**ECAR**	Extracellular Acidification Rate
**EGCG**	Epigallocatechin-3-gallate
**EGFR**	Epidermal growth factor receptor
**EMT**	Epithelial-Mesenchymal Transition
**ERK**	Extracellular Signal-Regulated Kinase
**FAP**	Familial Adenomatous Polyposis
**FoxM1**	Forkhead box M1
**FAS**	Fatty acid synthase
**FUT4**	Fucosyltransferase 4
**GA**	Gallic acid
**GGT**	r-glutamyltranspeptidase
**GADD45A**	DNA-Damage-Inducible 45 Alpha
**GPx**	Glutathione Peroxidase
**GST**	Glutathione-S-Transferase
**GSK3β**	Glycogen synthase kinase-3 beta
**GLUT1**	Glucose transporter 1
**HDAC**	Histone deacetylase
**HRK**	Harakiri
**HCC**	Hepatocellular Carcinoma
**HDM**	Histone Demethylase
**HIF-1α**	Hypoxia-Inducible Factor-1α
**HO**	Heme Oxygenase
**IL**	Interleukin
**IAP**	Inhibitors of apoptosis proteins
**INF**	Interferon
**IKKβ**	Factor-kappa-B kinase subunit beta
**IgM**	Immunoglobulin M
**IgG**	Immunoglobulin G
**JNK**	C-Jun N-Terminal Kinase
**JAK2**	Janus kinase 2
**LDH**	Lactate dehydrogenase
**MAPK**	Mitogen-Activated Protein Kinase
**MM**	Multiple Myeloma
**MMP**	Matrix Metalloproteinase
**MCP-1**	Monocyte chemoattractant protein-1
**MDR1**	Multidrug Resistance 1
**MUC1**	Mucin 1
**NF-κB**	Nuclear Factor Kappa B
**Nrf2**	Nuclear factor erythroid-related factor 2
**Notch-1**	Neurogenic locus notch homolog protein 1
**mTOR**	mammalian target of rapamycin
**NSCLC**	Non-Small Cell Lung Cancer
**OC**	Ovarian Cancer
**OCR**	Oxygen Consumption Rate
**PARP-1**	Poly (ADP-ribose) Polymerase-1
**PBMC**	Peripheral Blood Mononuclear Cell
**PEL**	Primary Effusion Lymphoma
**P-gp**	P-glycoprotein
**PI3-kinases**	Phosphoinositide 3-Kinase
**PSA**	Prostate-Specific Antigen
**PTEN**	Phosphatase and Tensin Homolog
**PTX**	Paclitaxel
**PKB**	Protein kinase B
**PKM-2**	Pyruvate kinase muscle-2
**p53**	Tumor protein 53
**PFKFB3**	Phosphofructo-2-kinase/fructose-2,6-biphosphatase 3
**PDHK1**	Pyruvate dehydrogenase kinase1
**RA**	Rosmarinic acid
**ROS**	Reactive Oxygen Species
**SA**	Sinapic acid
**SOD**	Superoxide Dismutase
**STAT3**	Signal Transducer and Activator of Transcription 3
***t*-BHP**	*tert*-Butyl HydroPeroxide
**TLR4**	Toll-Like Receptor 4
**TNFRSF11B**	TNF Superfamily 11B Receptor
**TNFRSF25**	Tumor Necrosis Factor Receptor Superfamily 25
**TNF-α**	Tumor Necrosis Factor-α
**TIMP-1**	Tissue inhibitor of metalloproteinase-1
**TGF-β**	Transforming growth factor beta
**TRIB3**	Tribbles homolog 3
**UDP-GT**	UDP-Glucuronyl Transferase
**VEGF**	Vascular Endothelial Growth Factor
**WT1**	Wilms Tumor Protein 1
**γCD**	Gamma-Cyclodextrin
**XIAP**	X-linked inhibitor of apoptosis
